# Critical role of caveolin-1 in aflatoxin B1-induced hepatotoxicity via the regulation of oxidation and autophagy

**DOI:** 10.1038/s41419-019-2197-6

**Published:** 2020-01-02

**Authors:** Qingqiang Xu, Wenwen Shi, Pan Lv, Wenqi Meng, Guanchao Mao, Chuchu Gong, Yongchun Chen, Youheng Wei, Xiaowen He, Jie Zhao, Hua Han, Mingxue Sun, Kai Xiao

**Affiliations:** 1 Lab of Toxicology and Pharmacology, Faculty of Naval Medicine, Naval Medical University, Shanghai, 200433 China; 2 State Key Laboratory of Genetic Engineering, Institute of Genetics, School of Life Sciences, Fudan University, Shanghai, 200438 China; 3 Origincell Technology Group Co., Ltd, 1118 Halei Road, Shanghai, 201203 China; 4 School of Medicine, Tongji University, 1239 Siping Road, Shanghai, China

**Keywords:** Apoptosis, Stress signalling, Hepatotoxicity

## Abstract

Aflatoxin B1 (AFB1) is a potent hepatocarcinogen in humans and exposure to AFB1 is known to cause both acute and chronic hepatocellular injury. As the liver is known to be the main target organ of aflatoxin, it is important to identify the key molecules that participate in AFB1-induced hepatotoxicity and to investigate their underlying mechanisms. In this study, the critical role of caveolin-1 in AFB1-induced hepatic cell apoptosis was examined. We found a decrease in cell viability and an increase in oxidation and apoptosis in human hepatocyte L02 cells after AFB1 exposure. In addition, the intracellular expression of caveolin-1 was increased in response to AFB1 treatment. Downregulation of caveolin-1 significantly alleviated AFB1-induced apoptosis and decreased cell viability, whereas overexpression of caveolin-1 reversed these effects. Further functional analysis showed that caveolin-1 participates in AFB1-induced oxidative stress through its interaction with Nrf2, leading to the downregulation of cellular antioxidant enzymes and the promotion of oxidative stress-induced apoptosis. In addition, caveolin-1 was found to regulate AFB1-induced autophagy. This finding was supported by the effect that caveolin-1 deficiency promoted autophagy after AFB1 treatment, leading to the inhibition of apoptosis, whereas overexpression of caveolin-1 inhibited autophagy and accelerated apoptosis. Interestingly, further investigation showed that caveolin-1 participates in AFB1-induced autophagy by regulating the EGFR/PI3K-AKT/mTOR signaling pathway. Taken together, our data reveal that caveolin-1 plays a crucial role in AFB1-induced hepatic cell apoptosis via the regulation of oxidation and autophagy, which provides a potential target for the development of novel treatments to combat AFB1 hepatotoxicity.

## Introduction

Aflatoxins are secondary metabolites with high toxicity produced by different strains of fungi, such as *Aspergillus flavus* and *Aspergillus parasiticus*, and are a common dietary contaminant all over the world, especially in tropical and subtropical regions^1^. Among the known aflatoxin derivatives, aflatoxin B1 (AFB1) possesses the highest toxic potential and is known to be a potent carcinogenic to human^2^. It has been proven that AFB1 has highly hepatotoxic, genotoxic, and immunotoxic effects, among other adverse health effects, which lead to acute or chronic hepatocellular injury of the liver^3^.

The toxic and carcinogenic effects of AFB1 are intimately linked to its biotransformation, during which AFB1 is able to stimulate cellular metabolism to generate reactive oxygen species (ROS) and free radicals^4^. In general, ROS at physiological levels play an important role in normal cellular signaling to induce adaptive responses. However, high levels of ROS lead to cell death by inducing oxidative damage to susceptible proteins and lipids^5^. Several studies have characterized AFB1-induced oxidative damage and its role in hepatotoxicity^6,7^. AFB1 can impair the antioxidant/prooxidant imbalance and elevate lipid peroxidation, resulting in the damage of biological molecules including lipids, proteins, and DNA in cellular systems^8^. The combination of these effects leads to the activation of a programmed cell death process or induces cells to produce potentially catastrophic genetic alterations, which depend on the dose and duration of AFB1 exposure^9,10^. A growing body of evidence has demonstrated that the detoxification of this toxin can generally be mediated by antioxidants, such as heme oxygenase-1 (HO-1), NAD(P)H/quinone oxidoreductase-1 (NQO1), and glutathione *S*-transferases (GSH), indicating the importance of oxidative stress regulation in AFB1-induced hepatotoxicity^11^. However, the precise mechanism of AFB1-induced oxidative damage is not well understood, especially the key molecules and pathways that participate in this process in response to AFB1 stimulation.

Caveolin-1 (Cav-1) is a major resident scaffolding protein constituent of caveolae, which are specialized plasma membrane structures^12^. Cav-1 has a variety of biological functions, including the regulation of cholesterol homeostasis, vesicular transport, and signal transduction events^13^. In the liver, Cav-1 modulates several molecular pathways leading to the regulation of hepatic lipid accumulation, lipid and glucose metabolism, mitochondrial biology, and hepatocyte proliferation^14^. Cav-1 thus acts as a regulator of hepatic function. Currently, several studies have implicated Cav-1 in the regulation of oxidation. For instance, Cav-1 deficiency protects against hyperoxia-induced oxidative lung injury via the upregulation of HO-1. The expression of HO-1 was shown to be markedly elevated in lung tissue or fibroblasts from Cav-1^−/−^ mice^15^. Cav-1 interacts with mineralocorticoid receptors (MRs) and forms an MR/Cav-1 complex in caveolae, which mediates a rapid signaling cascade of oxidation^16^. In addition, Cav-1 has been shown to be an endogenous inhibitor of nuclear erythroid 2 p45-related factor-2 (Nrf2), a major inducible cellular and tissue defense factor against oxidative stress^17^. Cav-1 interacts with Nrf2 and suppresses its transcriptional activity^18^. These studies indicate that Cav-1 plays a promotional role in oxidative stress regulation. In contrast, emerging evidence shows that Cav-1 is a potential oxidative stress-related target during oxidative stress-induced cancer initiation and development. For example, Cav-1 attenuates hydrogen peroxide-induced oxidative damage in lung carcinoma cells^19^. More importantly, multiple antioxidants have been shown to exert antitumor activities in cancer cells and protective activities in normal cells by modulating the Cav-1 pathway, indicating that Cav-1 may be an oxidative stress suppression target for cancer antioxidant prevention^20,21^. Consequently, the role of Cav-1 in oxidation regulation is conflicting. Cav-1 may be a multifunctional signaling hub, which allows it to regulate oxidation.

Considering the role of oxidative stress in mediating AFB1-induced hepatotoxicity and the relationship between Cav-1 and oxidative stress regulation, the present study aimed to evaluate the involvement of Cav-1 in the cytotoxicity and apoptosis of L02 cells following AFB1 exposure. We demonstrate for the first time that Cav-1 plays a crucial role in AFB1-induced hepatic cell apoptosis via the regulation of oxidation and autophagy.

## Results

### AFB1 exposure induces a cell viability decrease, oxidative stress, and apoptosis

**Fig. 1:**
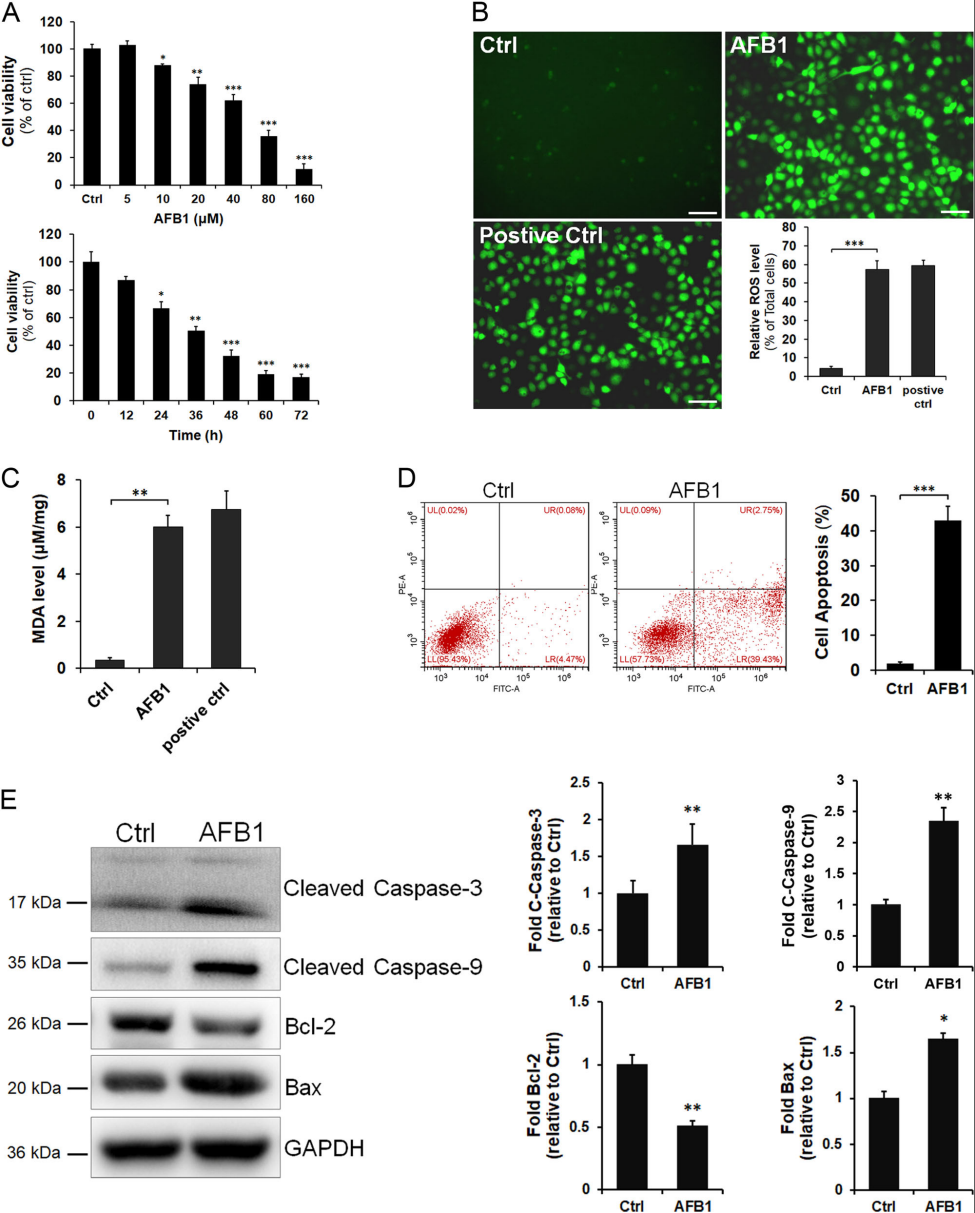
AFB1 exposure induces a decrease in L02 cell viability, oxidative stress, and apoptosis. **a** L02 cells were untreated (Ctrl) or treated with increasing concentrations of AFB1 (5, 10, 20, 40, 80, and 160 μM) for 24 h or with 40 μM AFB1 for the indicated time points (12, 24, 36, 48, 60, and 72 h); the cell viability was then detected by CCK-8 assay. **b** L02 cells were untreated (Ctrl) or treated with 40 μM AFB1 for 36 h. ROS production was detected by DCF probe staining and the percentage of DCF-positive hepatocytes was processed and quantified using ImageJ software (scale bar = 200 μm). **c** The levels of MDA were measured by detection kits after L02 cells were untreated or treated with 40 μM AFB1 for 36 h. **d** The induction of apoptosis in L02 cells was determined by Annexin V/PI flow cytometry following treatment with AFB1 (40 μM) for 36 h. Quantification of apoptotic cells is presented as the percent of total cells. **e** Western blotting analysis of apoptosis-related proteins in L02 cells treated with AFB1 (40 μM) for 36 h. The levels of cleaved caspase-3, cleaved caspase-9, Bcl-2, and Bax protein were quantified by densitometric analysis using ImageJ software and normalized to GAPDH. The data represent the mean ± SD of three independent experiments. *P < 0.05, **P < 0.01, and ***P < 0.001.

AFB1-induced hepatotoxicity was analyzed in human L02 cells because of their similarity to primary hepatic cells. This cell line has previously been widely used to study hepatotoxicity and hepatic injury^22,23^. To directly detect the cytotoxicity induced by AFB1, L02 cells were treated with increasing concentrations of AFB1 or treated with AFB1 for different durations, and the cell viability was then measured by Cell Counting Kit-8 (CCK-8) assay. As shown in Fig. 1a, AFB1 treatment significantly reduced cell viability in a dose- and time-dependent manner. The cell viability was reduced by approximately 50% in cultures treated with 40 μM AFB1 for 36 h compared with that in Ctrl cultures (**P < 0.01). Thus, 40 μM AFB1 treatment was used for subsequent experiments. To investigate the effect of AFB1 on the cell oxidative stress reaction, ROSs were measured in L02 cells using a DCF-DA probe. Imaging indicated that the percentage of DCF-positive cells increased after AFB1 treatment (Fig. 1b; ***P < 0.001). Malondialdehyde (MDA) levels were also increased in the AFB1 treatment group (Fig. 1c; **P < 0.01). Thus, these results indicate that AFB1 treatment can significantly induce oxidative stress in cells. Apoptosis can be induced by oxidative stress^24^. We found that the number of apoptotic cells increased compared with that of the Ctrl groups at 36 h after treatment (Fig. 1d; ***P < 0.001). Oxidative stress-induced apoptosis is usually associated with caspase activation^25^. Thus, we next measured the effect of AFB1 on the caspase-9 and caspase-3 pathways, and noted that the levels of cleaved caspase-9 and cleaved caspase-3 were increased at 36 h after AFB1 treatment (Fig. 1e). Taken together, these results indicate that AFB1 exposure induces oxidative stress and apoptosis, leading to a decrease in cell viability.

### AFB1 upregulates the expression of Cav-1 in hepatic cells

**Fig. 2:**
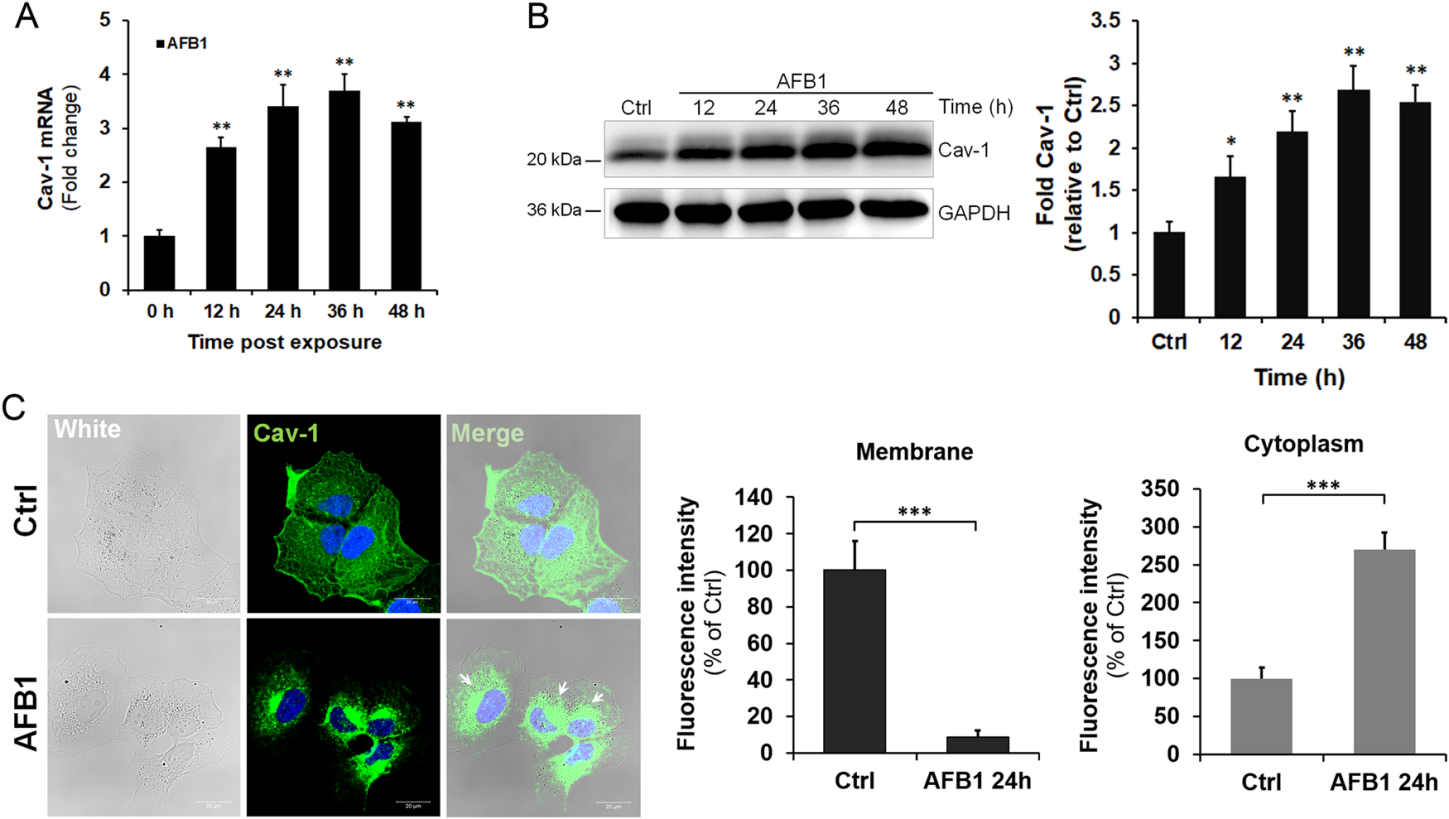
Cav-1 expression is upregulated in L02 cells after AFB1 stimulation. L02 cells were treated with AFB1 (40 μM). The cells were collected at different time points. **a** Cav-1 mRNA levels were determined by RT-qPCR at different time points. **b** The protein levels of Cav-1 were examined in untreated (Ctrl) or AFB1-treated (40 μM) L02 cells via western blotting. GAPDH served as a control for equal sample loading. The level of Cav-1 was quantified by densitometric analysis using ImageJ software and normalized to GAPDH. **c** Localization of Cav-1. L02 cells were fixed following treatment with AFB1 (40 μM) for 36 h and were subjected to immunofluorescence to detect Cav-1 (green). The nuclei were stained with DAPI (blue). Images were obtained using a confocal microscope (scale bar = 20 μm). **d** The fluorescence intensity of Cav-1 was processed and quantified using ZEN Light Edition software. The data are shown as the mean ± SD of three independent experiments. *P < 0.05, **P < 0.01, and ***P < 0.001.

To determine whether AFB1 treatment alters Cav-1 expression, L02 cells were treated with AFB1 at a concentration of 40 μM and then Cav-1 mRNA levels were determined by reverse-transcriptase quantitative PCR (RT-qPCR) at different time points. As shown in Fig. 2a, Cav-1 mRNA levels were upregulated after AFB1 treatment. Moreover, Cav-1 protein levels were examined after AFB1 treatment by western blotting and the results showed that Cav-1 levels were significantly increased in AFB1-treated cells at 24 h, 36 h, and 48 h after treatment (Fig. 2b; **P < 0.01). Furthermore, immunofluorescent staining was performed to determine whether AFB1 treatment alters Cav-1 subcellular localization. As shown in Fig. 2c, Cav-1 staining was more apparent in the perinuclear region of AFB1-treated cells compared with untreated cells when examined at 36 h after treatment. The Cav-1 fluorescence intensity was decreased in the membrane and increased in the cytoplasm of treated cells (Fig. 2d). These data indicate that AFB1 treatment upregulates the expression of Cav-1 and promotes the aggregation of Cav-1 in the perinuclear region of the cell.

### Cav-1 plays an important role in the regulation of AFB1-induced hepatotoxicity

**Fig. 3:**
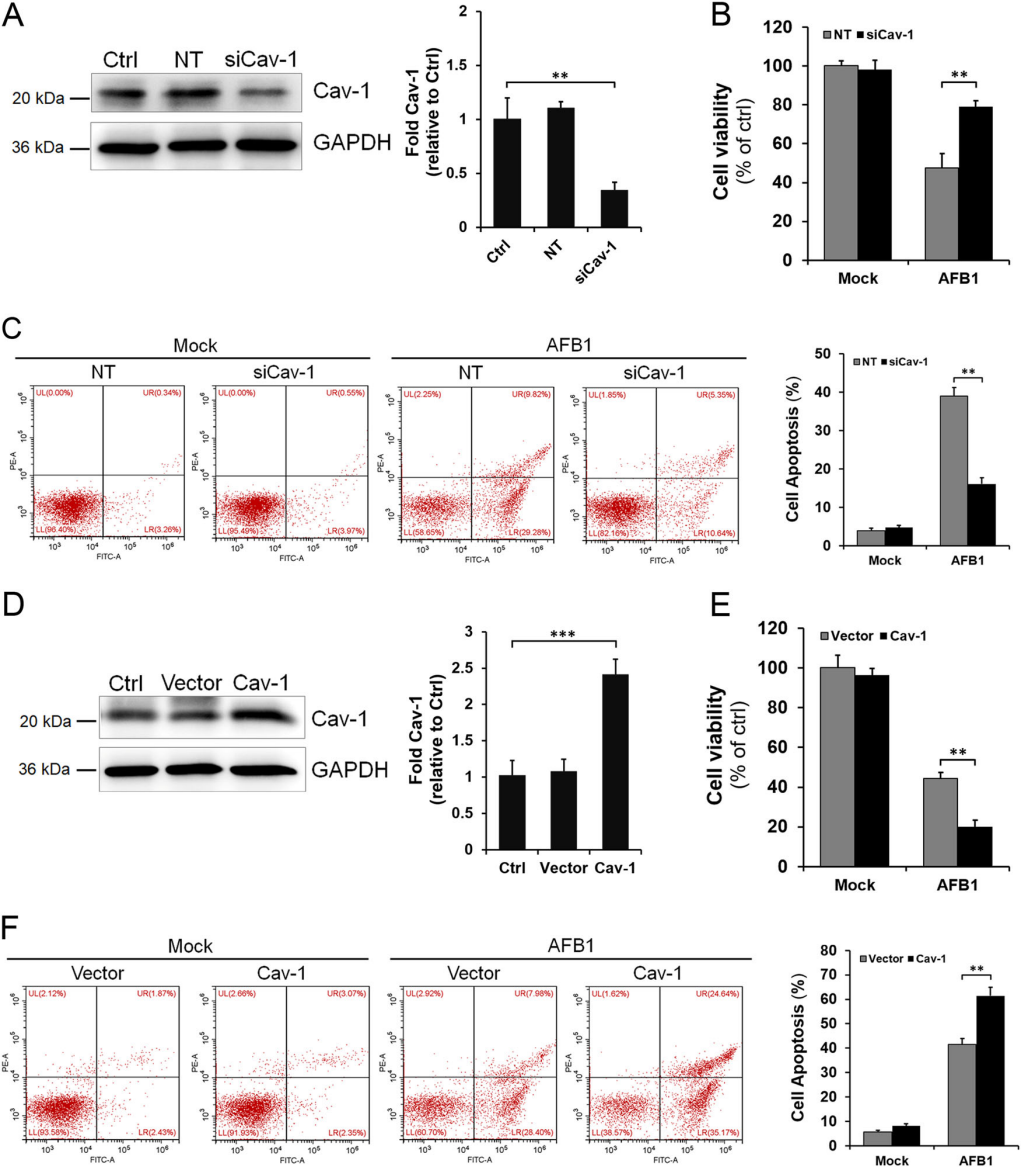
Cav-1 plays an important role in the regulation of AFB1-induced hepatotoxicity. L02 cells were transfected with Cav-1-specific siRNA (100 nM) or the non-target (NT) siRNA (100 nM) for 72 h. **a** The reduction of Cav-1 protein level after treatment with siRNA was detected by western blotting, quantified by densitometric analysis using ImageJ software, and normalized to GAPDH. **b** Cells transfected with Cav-1 siRNA or NT siRNA were treated with AFB1 (40 μM) and cell viability was then detected by CCK-8 assay. **c** The induction of apoptosis was determined by Annexin V/PI flow cytometric analysis following treatment with AFB1 (40 μM) for 36 h. Quantification of apoptotic cells is presented as the percent of total cells. **d**–**f** L02 cells were transfected with GFP-tagged constructs expressing Cav-1 or the empty vector for 48 h. **d** The Cav-1 protein level was detected by western blotting, quantified by densitometric analysis using ImageJ software, and normalized to GAPDH. Cell viability was detected by CCK-8 assay (**e**) and the induction of apoptosis was determined by Annexin V/PI flow cytometry (**f**) following treatment with AFB1 (40 μM) for 36 h. The data are shown as the mean ± SD of three independent experiments. *P < 0.05, **P < 0.01, and ***P < 0.001.

To determine the role of Cav-1 in the regulation of AFB1-induced hepatotoxicity, L02 cells were transfected with Cav-1 small interfering RNA (siRNA) or non-target (NT) siRNA and then treated with 40 μM AFB1 at 72 h post transfection. The depletion of Cav-1 in L02 cells was confirmed by western blotting (Fig. 3a). Cell viability was then measured at 36 h after treatment. As shown in Fig. 3b, Cav-1 siRNA significantly alleviated the AFB1-induced cell viability decrease compared with the NT siRNA. Moreover, AFB1-induced apoptosis was significantly inhibited when Cav-1 was knocked down (Fig. 3c; **P < 0.01), indicating hepatotoxicity inhibition in Cav-1-deficient cells. We next sought to determine whether Cav-1 overexpression had an adverse effect in the context of AFB1 treatment. As shown in Fig. 3e, f, the increase in Cav-1 expression significantly improved AFB1-induced hepatotoxicity and apoptosis, indicating that Cav-1-overexpressing cells were more susceptible to AFB1-induced cell death, which is consistent with the results in Cav-1-deficient cells shown in Fig. 3b, c. The overexpression of Cav-1 in L02 cells was confirmed by western blotting (Fig. 3d). Taken together, the above results suggest that Cav-1 plays an important role in the regulation of AFB1-induced hepatotoxicity.

### Cav-1 regulates AFB1-induced oxidative stress and apoptosis through its interaction with Nrf2

**Fig. 4:**
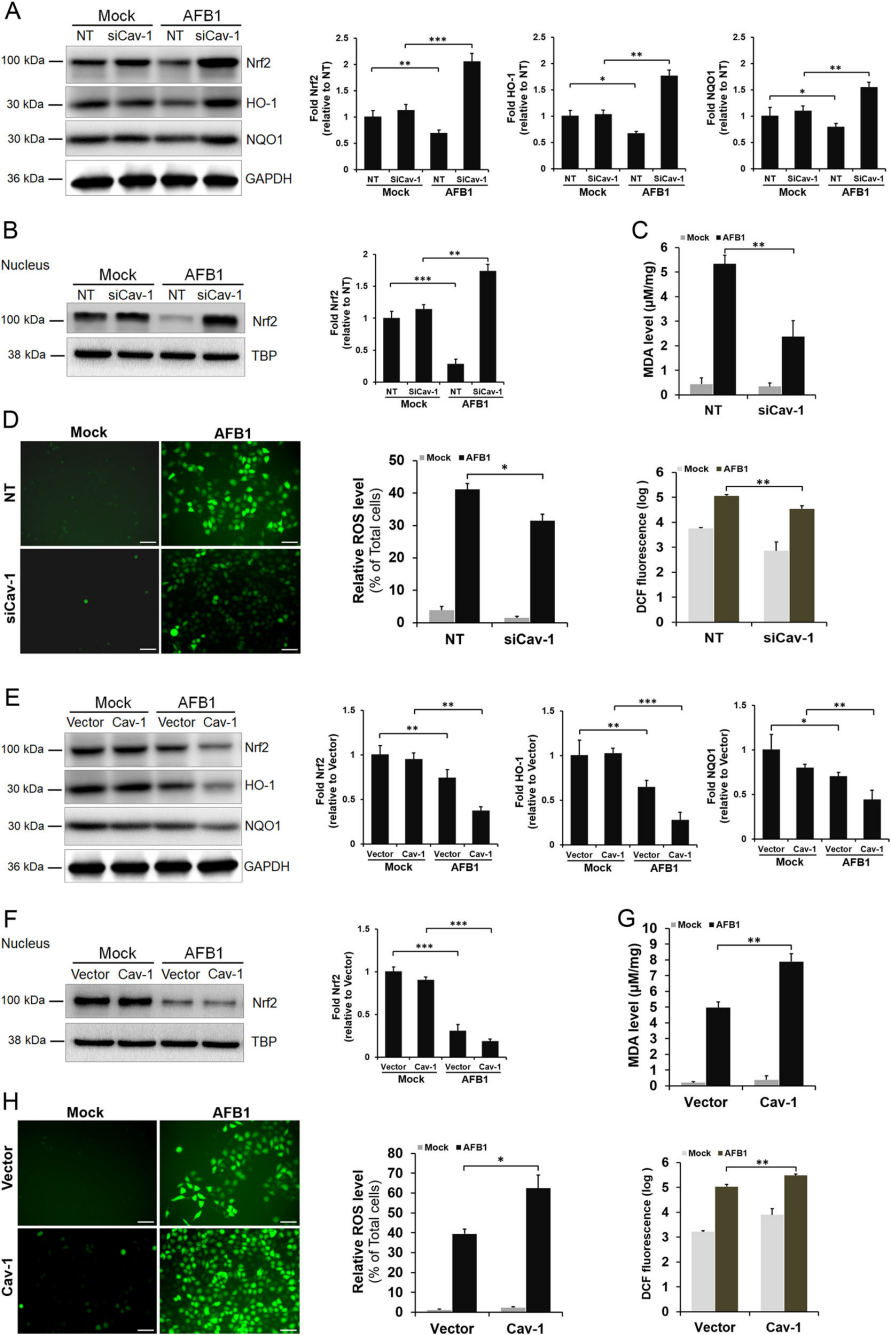
Cav-1 regulates AFB1-induced oxidative stress through the Nrf2 pathway. Cells transfected with Cav-1 siRNA or the NT siRNA were mock-treated or treated with AFB1 (40 μM) for 36 h. **a** Cells were lysed at the indicated time points and immunoblotted with anti-Nrf2, anti-HO-1, and anti-NQO1 antibodies. The protein expression level was quantified by densitometric analysis using ImageJ software and normalized to GAPDH. **b** Cells were collected for the isolation of nuclear proteins and used for the detection of nuclear Nrf2 protein levels. The protein expression level was quantified by densitometric analysis using ImageJ software and normalized to TBP. **c** Levels of MDA were measured by detection kits. **d** ROS production was detected by DCF probe staining, the percentage of DCF-positive hepatocytes, and the fluorescence intensity was processed and quantified using ImageJ software. Cells transfected with GFP-tagged constructs expressing Cav-1 or the empty vector were mock-treated or treated with AFB1 (40 μM) for 36 h. **e**, **f** The Nrf2, HO-1 NQO1, and nuclear Nrf2 protein levels were detected by western blotting, quantified by densitometric analysis using ImageJ software, and normalized to GAPDH or TBP. MDA levels were measured by a detection kit (**g**) and ROS production was detected by DCF probe staining (**h**). Scale bar = 200 μm. The data are shown as the mean ± SD of three independent experiments. *P < 0.05, **P < 0.01, and ***P < 0.001.

As AFB1 exposure induces oxidative stress and Nrf2 is a central protein in the regulation of the cellular antioxidant response, we explored whether Cav-1 regulates AFB1-induced oxidative stress and apoptosis through the regulation of the Nrf2 pathway. L02 cells were transfected with Cav-1 siRNA or NT siRNA and then treated with or without AFB1. The expression levels of Nrf2 and the antioxidant enzymes HO-1 and NQO1 were measured 36 h after AFB1 treatment. As shown in Fig. 4a, Cav-1 depletion did not significantly affect the Nrf2, HO-1, and NQO1 protein levels in mock-treated cells; however, these protein levels were markedly enhanced in Cav-1 siRNA-transfected cells relative to those in NT-transfected cells after AFB1 treatment, indicating that Cav-1 depletion leads to increased Nrf2 and antioxidant enzyme expression levels in response to AFB1 treatment. Nrf2 need to translocate into the nucleus where it binds the antioxidant response element (ARE), leading to transcription of downstream genes^26^. The Nrf2 protein levels in the nuclear extractions were further detected. Compared with NT treatment, Cav-1 depletion slightly promoted Nrf2 nucleus translocation in mock-treated cells, but without statistical significance. However, Nrf2 protein levels in the nuclear were markedly enhanced in Cav-1-depleted cells relative to those in NT-transfected cells after AFB1 treatment (Fig. 4b). Furthermore, Cav-1 knockdown also attenuated AFB1-induced MDA and ROS levels (Fig. 4c, d). However, these effects were reversed when Cav-1 was overexpressed in L02 cells. As shown in Fig. 4d, Cav-1 overexpression reduced Nrf2, HO-1, and NQO1 protein levels. The ROS and MDA levels increased markedly as the response increased (Fig. 4e–h).

**Fig. 5:**
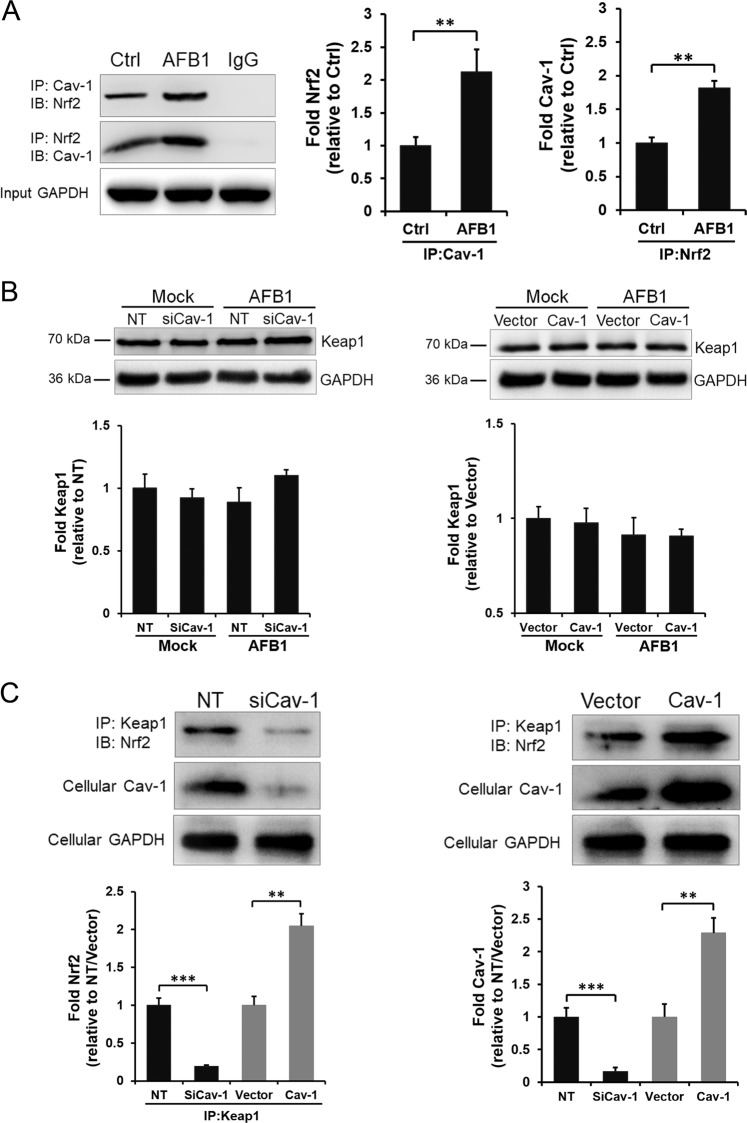
Role of Cav-1 in the regulation of Nrf2 transcriptional activity and the Keap1–Nrf2 interaction. **a** L02 cells were untreated or treated with AFB1 (40 μM) for 36 h and collected samples were immunoprecipitated (IP) and immunoblotted (IB) with the indicated antibodies using the “Tag-Switch” method. Nonspecific IgG served as an input control. L02 cells transfected with NT, Cav-1 siRNA, Cav-1 plasmid, and the vector control were untreated or treated with AFB1 (40 μM) for 36 h, (**b**) cells were lysed and immunoblotted with anti-Keap1 antibodies, and (**c**) samples were collected for immunoprecipitation (IP) or western blotting (IB) analysis as indicated. The protein expression level was quantified by densitometric analysis using ImageJ software and normalized to GAPDH. The data are shown as the mean ± SD of three independent experiments. *P < 0.05, **P < 0.01, and ***P < 0.001.

To explore the mechanisms of the Cav-1 in the regulation of Nrf2 pathway, the interaction between Cav-1 and Nrf2 was examined by immunoprecipitation. As shown in Fig. 5a, Cav-1 and Nrf2 constitutively interacted with each other under basal conditions, and the association between Cav-1 and Nrf2 was enhanced after the stimulation with AFB1. As the Keap1–Nrf2 system plays a central role in the regulation of the levels of cellular antioxidants such as HO-1 and GCLC^27^, we further explored the role of Cav-1 in the Keap1–Nrf2 interaction. The Keap1 mRNA transcription level and protein level under AFB1 treatment were first detected. As shown in Fig. 5b and Supplementary Fig. S1, Cav-1 downregulation had no significant effect on Keap1 expression in both vehicle and AFB1-treated samples. Interestingly, Cav-1 knockdown eventually decreased the Keap1–Nrf2 interaction after stimulation with AFB1 (Fig. 5c, left panels), whereas overexpression of Cav-1 facilitated the Keap1–Nrf2 association (Fig. 5c, right panels), indicating that Cav-1 mediates the Keap1–Nrf2 interaction in response to AFB1 stimulation, which is consistent with the results of the Nrf2 and antioxidant protein expression shown in Fig. 4a, e.

### Cav-1 promotes AFB1-induced apoptosis by inhibiting autophagy

**Fig. 6:**
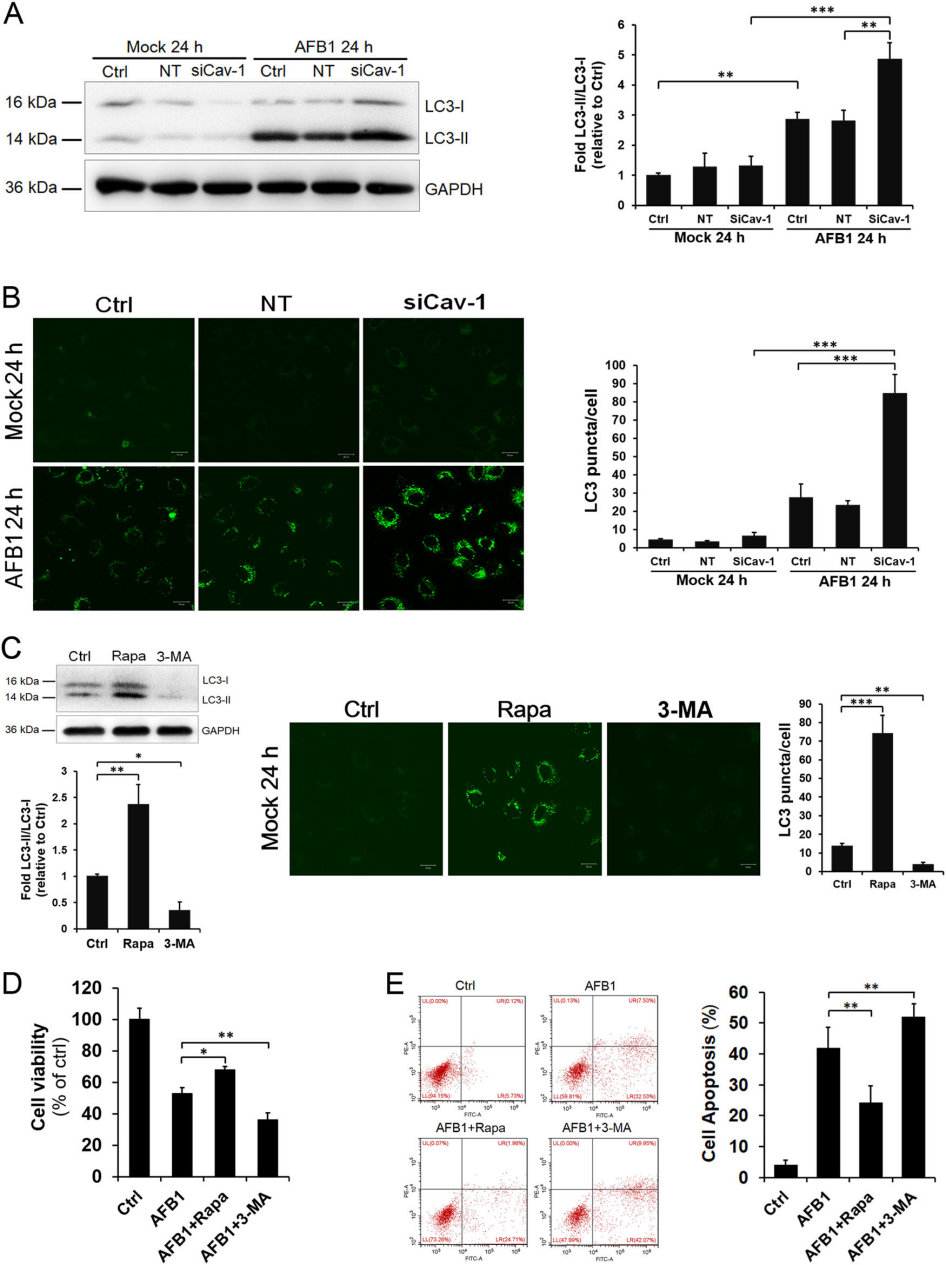
Cav-1 promotes AFB1-induced apoptosis by inhibiting autophagy. L02 cells transfected with Cav-1 siRNA or the NT siRNA were mock-treated or treated with AFB1 (40 μM). **a** The protein levels of LC3-I and LC3-II were determined by western blotting at 24 h after AFB1 treatment. GAPDH served as a control for equal sample loading. The relative quantification of the detected signal was analyzed using ImageJ software and normalized to GAPDH. **b** Representative fluorescent images and statistical results of LC3 puncta at 24 h after AFB1 treatment. Cells with autophagosomes were chosen from a pool of at least ten images. The scale bar is 20 µm. **c** Cells were pretreated with rapamycin (200 nM) or 3-MA (10 mM) for 1 h and then incubated with AFB1 in the presence of inhibitors. The protein levels of LC3-I and LC3-II were determined by western blotting and representative fluorescent images of LC3 puncta were analyzed at 24 h after AFB1 treatment. **d**, **e** Cells were pretreated with rapamycin (200 nM) or 3-MA (10 mM) for 1 h and then incubated with AFB1 in the presence of inhibitors. Cell viability was detected by CCK-8 assay (**d**) and the induction of apoptosis was determined by Annexin V/PI flow cytometry (**e**) following treatment with AFB1 (40 μM) for 36 h. The data are shown as the mean ± SD of three independent experiments. *P < 0.05, **P < 0.01, and ***P < 0.001.

Autophagy is an intracellular lysosomal degradation pathway, the primary function of which is to allow cells to survive under various stresses. In the liver, autophagy attenuates the aggregation of abnormal protein and protects hepatocytes against injury^28,29^. Previous studies have shown that autophagy can be regulated by Cav-1^30^. To investigate the possible relationship between Cav-1 and the autophagic pathway in response to AFB1 stimulation, Cav-1 was depleted by RNA interference and then autophagosome accumulation was detected. Cav-1 depletion did not increase the LC3-II levels in mock-treated cells (Fig. 6a); however, the LC3-II levels were significantly enhanced in Cav-1 siRNA-transfected cells compared with NT-transfected cells after AFB1 exposure (Fig. 6a), indicating that Cav-1 depletion leads to increased LC3-II accumulation. These observations were further confirmed by detecting the distribution of GFP-LC3 puncta, which represent autophagosomes. AFB1 stimulation leads to accumulation of autophagosomes in cells as detected by GFP-LC3 punctate distribution. There were 70–100 GFP-LC3 puncta per cell in Cav-1 siRNA-transfected cells compared with 10–40 in NT-transfected cells under AFB1 treatment (Fig. 6b).

As autophagy has been shown to be linked with AFB1-induced apoptosis^31–33^, L02 cells were pretreated with rapamycin, an inducer of autophagy, or 3-Methyladenine (3-MA), an inhibitor of autophagy, to further confirm its role. The autophagy regulation function of rapamycin and 3-MA was confirmed by detecting LC3-II accumulation and LC3 puncta distribution (Fig. 6c). As shown in Fig. 6d, e, rapamycin treatment significantly reduced the AFB1-induced cell viability decrease and apoptosis, whereas 3-MA treatment significantly promoted the cell viability decrease and apoptosis. Taken together, these results suggest that Cav-1 negatively regulates autophagy after AFB1 stimulation, which facilitates apoptosis.

### Cav-1 mediates autophagy inhibition in response to AFB1 treatment via regulation of the EGFR/PI3K/mTOR pathway

**Fig. 7:**
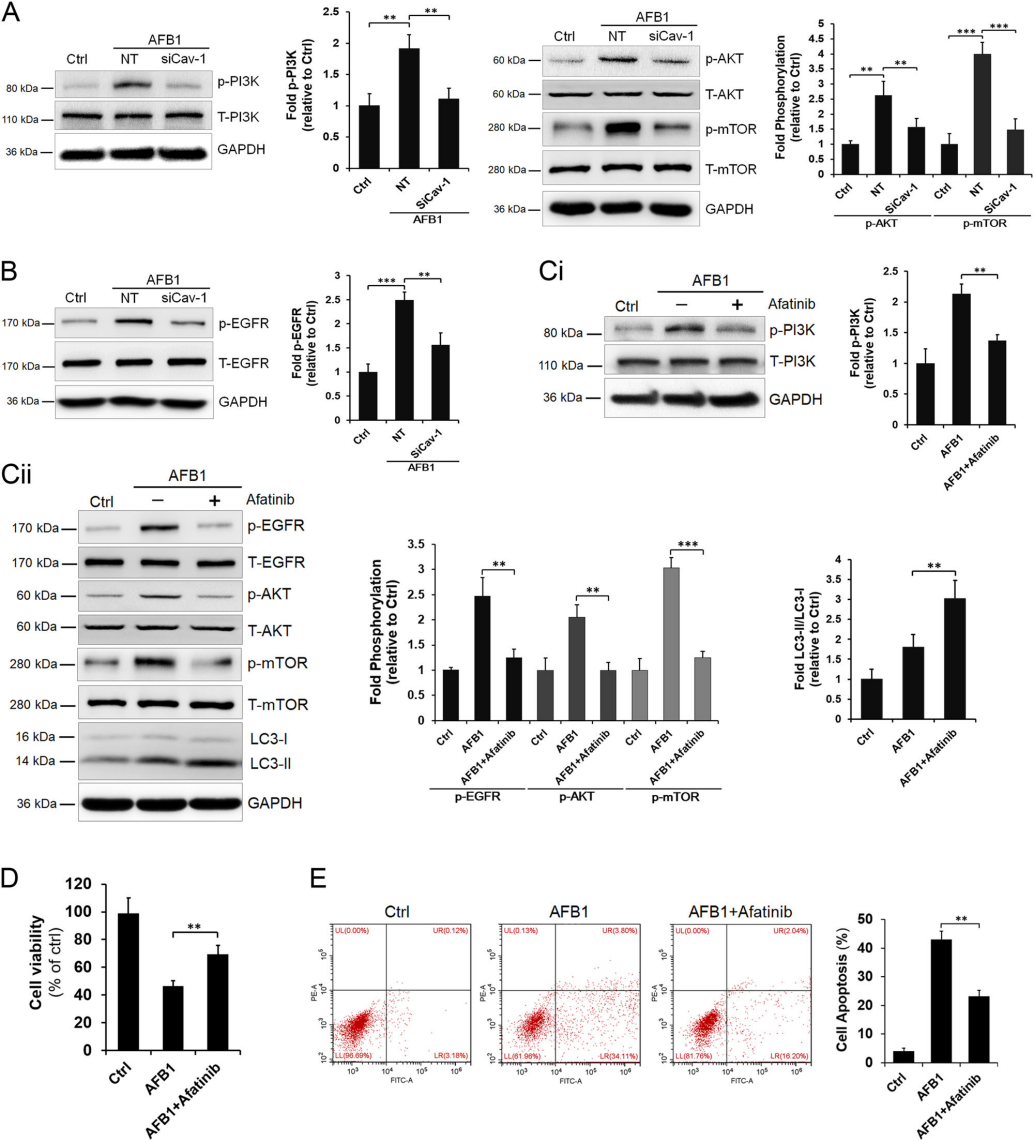
Cav-1 mediates autophagy inhibition in response to AFB1 treatment via regulation of the EGFR/PI3K/mTOR pathway. L02 cells transfected with Cav-1 siRNA or NT siRNA were untreated or treated with AFB1 (40 μM). The protein levels of (**a**) phosphorylation of PI3K, total PI3K, phosphorylation of AKT, total AKT, phosphorylation of mTOR, total mTOR, and (**b**) phosphorylation of EGFR and total EGFR were determined by western blotting at 36 h after AFB1 treatment. GAPDH served as a control for equal sample loading. The relative quantification of the detected signal was analyzed using ImageJ software and normalized to GAPDH. **c**–**e** Cells were pretreated with afatinib (10 μM) for 1 h and then incubated with AFB1 in the presence of the inhibitor. The cell lysates were analyzed for the phosphorylation of PI3K, EGFR, AKT, mTOR, and LC3-I/LC3-II by western blotting (**c**). Cell viability was detected by CCK-8 assay (**d**) and the induction of apoptosis was determined by Annexin V/PI flow cytometry (**e**) following treatment with AFB1 (40 μM) for 36 h. The data are shown as the mean ± SD of three independent experiments. *P < 0.05, **P < 0.01, and ***P < 0.001.

Cav-1 has been shown to negatively or positively regulate autophagy through interactions with ATG5, ATG7, or beclin-1, which are key components in the formation of autophagosomes^34^. We found that whether Cav-1 depletion or overexpression did not affect the expression of ATG5, ATG7, and beclin-1 in response to AFB1 stimulation (Supplementary Fig. S2). However, the phosphoinositide 3-kinase (PI3K)-AKT/mammalian target of rapamycin (mTOR) pathway, which is the canonical autophagy regulation pathway, was activated in response to AFB1 treatment (Fig. 7a). Phosphorylation of PI3K, AKT, and mTOR was significantly reduced when Cav-1 was depleted by RNA interference, indicating that Cav-1 depletion inhibits AFB1-induced PI3K-AKT/mTOR pathway activation. As epidermal growth factor receptor (EGFR), the direct upstream kinase of PI3K/AKT, is a member of the RTK family, and several studies have demonstrated that EGFR is a critical regulator of autophagy^35,36^, we subsequently investigated the role of EGFR in PI3K-AKT/mTOR signaling after AFB1 stimulation. As shown in Fig. 7b, AFB1 treatment promoted EGFR phosphorylation; however, Cav-1 depletion noticeably reduced the level of phosphorylated EGFR. Furthermore, inhibition of the EGFR kinase by the EGFR-specific inhibitor afatinib inhibited the phosphorylation of PI3K, AKT, and mTOR, and increased the level of LC3, further confirming the role of EGFR in the regulation of autophagy in response to AFB1 stimulation (Fig. 7c). Moreover, afatinib treatment significantly reduced the AFB1-induced cell viability decrease and apoptosis (Fig. 7d, e). These results suggest that Cav-1 inhibits autophagy by regulating the EGFR/PI3K-AKT/mTOR pathway during AFB1 stimulation.

## Discussion

The hepatotoxicity induced by AFB1 has been well documented. It has been suggested that AFB1 exposure is associated with severe liver injury or the development of hepatocellular carcinoma^3^. However, the precise mechanism of AFB1-induced hepatotoxicity remains unclear and a specific treatment to inhibit hepatocellular damage caused by AFB1 is not available. In this study, an important role of Cav-1 in AFB1-induced hepatotoxicity was identified. Our findings show that (1) AFB1 upregulates the expression of Cav-1 in hepatic cells. (2) Cav-1 upregulation promotes AFB1-induced oxidative stress and apoptosis through direct interaction with Nrf2. (3) Cav-1 upregulation promotes AFB1-induced apoptosis by inhibiting autophagy via the regulation of the EGFR/PI3K/mTOR pathway. These findings suggest that Cav-1 plays a crucial role in AFB1-induced hepatic cell apoptosis via the regulation of oxidation and autophagy.

Several in vitro studies have demonstrated that AFB1-induced hepatotoxicity is predominantly exerted upon activation by its biotransformation in the liver. AFB1 requires metabolic activation by the cytochrome P450 (CYP-450) enzyme system to generate AFB1-exo-8,9-epoxide (AFBO) and then exert its cytotoxic and carcinogenic effects. AFBO and other metabolites interact with various biomolecules in the cell, including nucleic acids and proteins^4^. Moreover, AFBO accumulation depletes GSH due to the formation of large amounts of epoxides and other ROS, leading to apoptosis, cytotoxicity, and genotoxicity in human hepatocytes^8,37^. In our study, L02 cells exhibited increased production of ROS and MDA when exposed to AFB1, indicating the presence of oxidative stress. Furthermore, apoptosis of cells was significantly increased by the activation of caspases (Fig. 1). There is now accumulating evidence indicating that caspase activation is a common signaling process during ROS-induced cell death^24,38^. Increased caspase-3/9 activation and Bax expression has been observed in response to AFB1 treatment, which is indicative of apoptosis triggered through the mitochondrial signaling pathways^25,39^. Consequently, these results suggest that oxidative stress generated after AFB1 exposure is one of the main causes of hepatic cell injury and regulates cell apoptosis.

As a crucial factor, Cav-1 connects with liver function regulation^12,14^. Findings regarding the effects of Cav-1 on liver physiology and disease are controversial. For instance, deletion of Cav-1 attenuates lipopolysaccharide and d-galactosamine-induced acute liver injury in mice by decreasing the expression of TLR4, CD14, and adhesion molecules^40^; however, other data show that deletion of Cav-1 increases mortality and the production of inflammatory cytokines and NO in mice infected with *Salmonella enterica* serovar Typhimurium^41^, indicating that Cav-1 may play different roles when faced with different stimuli in the liver. Here we found that AFB1 treatment promoted the expression of Cav-1 in hepatocytes and Cav-1 downregulation significantly alleviated AFB1-induced apoptosis and the decrease in cell viability, suggesting that Cav-1 acts as an effector in response to AFB1 and participates in AFB1-induced hepatotoxicity. In addition, AFB1 treatment promoted the aggregation of Cav-1 in the perinuclear region of the cell, indicating that Cav-1 may function in that subcellular area during AFB1 stimulation. Although Cav-1 is the principal scaffolding protein of caveolae in the cell membrane, it can be internalized into the intracellular region. Moreover, compared with the limited distribution on the plasma membrane of caveolae^12^, Cav-1 also has an extensive intracellular membrane distribution including being distributed on mitochondria and the endoplasmic reticulum, as well as on late endosomes/lysosomes, indicating the existence of caveolae-independent functions of Cav-1 in the intracellular membrane system^42,43^.

Recently, increasing evidence has shown that Cav-1 is an oxidation-related protein. However, the role of Cav-1 in oxidation regulation is conflicting. It is reported that Cav-1 is necessary for hepatic oxidative lipid metabolism via the nuclear hormone receptor (PPARα, FXRα, and SHP) and bile acid signaling^44^. Cav-1^−/−^ endothelial cells display an enhanced antioxidant response. Reduction of Cav-1 shows a protective effect against polychlorinated biphenyl-induced cellular dysfunction and inflammation via the promotion of antioxidant gene expression^26^. Nevertheless, Cav-1 has been shown to act as a negative regulator of NOX-derived ROS through direct binding and alteration of its expression^45^. Pavlides et al.^46^ demonstrated that Cav-1 loss could induce oxidative stress, mimic hypoxia, and drive inflammation in the tumor microenvironment. These results indicate that the effects of Cav-1 on redox modification may vary by cell type. Our results demonstrate that Cav-1 positively regulates AFB1-induced oxidative stress by inhibiting the expression of antioxidant enzymes in hepatocytes. The increased expression of Cav-1 in response to AFB1 stimulation elevated the ROS and MDA levels, leading to apoptosis. Furthermore, we found that Cav-1 negatively regulated the expression of antioxidant enzymes through the Nrf2 pathway. Activated Nrf2 dissociates from its repressor protein Keap1 and then interacts with AREs. This process induces the subsequent expression of numerous downstream genes, such as *HO-1* and *NQO1*, and finally restores redox homeostasis^27,47^. A previous study showed that Silencing Cav-1 decreased both mRNA and protein levels of Nrf2 inhibitory protein Keap1 in endothelial cells, resulting in higher mRNA levels of the antioxidant genes^26^. However, it was found that Cav-1 downregulation had no significant effect on Keap1 expression in both vehicle and AFB1-treated samples in our study. Similar to the dual effect of Cav-1 in oxidative stress regulation, the oxidative stress regulation pathways may also diverse. Cav-1 has proven to be a direct binding partner of Nrf2 via the caveolin-binding domain (amino acids 281–289)^17^. Our results demonstrate that Cav-1 can directly interact with Nrf2, thereby affecting the Keap1–Nrf2 association and nucleus translocation of Nrf2. Therefore, Cav-1 could act as an inhibitor of Nrf2 and suppress its activity, resulting in the attenuation of cellular antioxidant capacity.

Autophagy is a major protective pathway that is activated in cells to facilitate adaption to various stresses^48,49^. Recent studies have reported the modulation of autophagy pathways by mycotoxin in different cellular models^50^. Currently, several studies have suggested that oxidative stress is linked with autophagy and apoptosis via Nrf2 pathway^28,31,51–55^. Moreover, Cav-1 has been shown participates in the regulation of autophagy under several stress conditions. Loss of Cav-1 promotes autophagy during hypoxia and oxidative stress in adipocytes and fibroblasts^30^. Cav-1-related autophagy initiated by aldosterone-induced oxidation can promote liver sinusoidal endothelial cell defenestration^56^. In our study, although autophagy was induced in response to AFB1 treatment, enhanced autophagosome accumulation was observed in Cav-1-depleted cells, suggesting that Cav-1 negatively regulates autophagy during AFB1 stimulation.

The mechanisms of Cav-1-mediated autophagy regulation are complicated. It has previously been reported that intracellular Cav-1 mediates autophagy through the regulation of the ATG12-ATG5 system^57^. In addition, Cav-1 can negatively regulate autophagy under basal or nutrient-deprivation conditions^58^. It has also been shown that Cav-1 interacts with beclin-1 to initiate autophagy^34^. Therefore, the ways by which Cav-1 regulates autophagy might be context-dependent. Previous studies showed that AFB1-induced autophagy regulates the major autophagy markers, such as beclin-1, ATGs, LC3-II, and p62, in macrophages^32^. We found that AFB1 stimulation did not affect ATG5, ATG7, and beclin-1 expression; nevertheless, the PI3K-AKT/mTOR pathway, which acts as the canonical pathway negative regulation pathway of autophagy, was activated. Furthermore, we found that Cav-1 did not interact with PI3K-AKT/mTOR directly but via the regulation of EGFR activity. As a molecular hub, Cav-1 can integrate the transduction and regulation of a variety of signaling molecules, including mitogen-activated protein kinases, EGFR, and transforming growth factor-β. The interactions between Cav-1 and these molecules occur through the Cav-1-scaffolding domain in the Cav-1 protein^13^. Therefore, we identified a nonclassical autophagy regulatory pathway in the present study and our results suggest that Cav-1 inhibits autophagy by regulating the EGFR/PI3K-AKT/mTOR pathway during AFB1 stimulation. However, it remains unclear how Cav-1 associates with EGFR to regulate autophagy. As Cav-1 and EGFR are all located in the lipid raft domain of lipid bilayer membrane structures^36^, it will be interesting to determine whether lipid rafts act as a signaling platform for the Cav-1 and EGFR interaction.

In conclusion, the present study reveals that Cav-1 plays a crucial role in AFB1-induced hepatic cell apoptosis via the regulation of oxidation and autophagy. Understanding the mechanism of Cav-1 in AFB1-induced hepatotoxicity will be beneficial for the development of new strategies to prevent or alleviate AFB1-induced hepatotoxicity.

## Materials and methods

### Reagents and antibodies

The following chemicals and antibodies were used in our experiments: AFB1 (A606874) was obtained from Sangon Biotech (Shanghai). 3-MA (#S2767), rapamycin (#S1039), and afatinib (#S1011) were purchased from Selleck Chemicals. DALGreen-Autophagy Detection Probe (#D675) was purchased from Dojindo Laboratories. Antibody against Cav-1 (ab2910) was purchased from Abcam. Antibodies against cleaved caspase-3 (#9664), cleaved caspase-9 (#20750), Bcl-2 (#15071), Bax (#2772), LC3B (#3868), Atg5 (#12994), Atg7 (#8558), beclin-1 (#3495), PI3K (#4249), phospho-PI3K (#4228), Akt (#4691), phospho-Akt (#4060), mTOR (#2983), phospho-mTOR (#5536), EGFR (#4267), phospho-EGFR (#3777), isotype control IgG (#3900), and glyceraldehyde-3-phosphate dehydrogenase (GAPDH) (#2118) were obtained from Cell Signaling Technologies. Antibodies against Nrf2 (16396-1-AP), HO-1 (10701-1-AP), NQO1 (11451-1-AP), Keap1 (10503-2-AP), and TBP (22006-1-AP) were purchased from Protein Tech. Horseradish peroxidase (HRP)-conjugated secondary antibodies against mouse (Catalog number 31430) or rabbit (Catalog number 31460) IgG, and Alexa Fluor 488-conjugated goat anti-rabbit IgG (Catalog number A-11034) were purchased from Life Technologies. siRNA targeting Cav-1 (L-003467-00-0005) and a non-targeting siRNA (D-001810-10-05) were purchased from Dharmacon. The green fluorescent protein-tagged constructs expressing wild-type Cav-1 and the vector were kindly provided by J. M. Bergelson (University of Pennsylvania)^59^.

### Cell culture

Human L02 cells were purchased from the Chinese Academy of Sciences and were maintained in RPMI 1640 containing 10% fetal bovine serum (FBS) (GIBCO) at 37 °C with 5% CO_2_. Hepatocellular carcinoma cell lines Huh7 and Hep G2 (ATCC, HB-8065) were cultured in Dulbecco’s modified Eagle’s medium supplemented with 10% FBS, 100 U/mL penicillin, and 100 μg/mL streptomycin (GIBCO). All cells were tested for mycoplasma contamination.

### Cell viability assay

The effect of AFB1 on cell viability was determined using the CCK-8 (CK04, Dojindo Laboratories) according to the manufacturer’s instructions. Briefly, cells (4 × 10^4^ cells/cm^2^) seeded in 96-well plates were incubated with AFB1 after the indicated pretreatments. The CCK-8 assay was performed by adding 110 μl of fresh medium containing 10 μl of CCK-8 solution to the cells and incubating them for 1–4 h at 37 °C. The light absorbance of the solution was measured at 450 nm by a microplate reader (BioTek).

### ROS measurement

The production of cellular ROS was measured with a Reactive Oxygen Species Assay kit (Beyotime) according to the manufacturer’s instructions. Briefly, the treated cells were incubated with DCF-DA (10 mM) for 30 min at 37 °C. After washing with phosphate-buffered saline (PBS), DCF-positive cells and intracellular fluorescence were observed with an Olympus fluorescence microscope (×200) and were quantified with ImageJ software.

### Malondialdehyde measurement

The treated cells were collected by trypsinization and then lysed in radioimmunoprecipitation assay (RIPA) buffer (Pierce). The lysate was then centrifuged at 12,000 × *g* for 10 min at 4 °C to collect the supernatant. Total protein concentrations of parallel samples were measured using a BCA Protein Assay kit (Beyotime). MDA levels were measured by using a Lipid Peroxidation MDA assay kit according to the manufacturer’s protocol (Beyotime).

### Apoptosis analysis

For quantification of apoptosis, the treated cells were collected and subjected to annexin V/propidium iodide (PI) double staining (BD) and were analyzed by using a flow cytometer (Beckman Cytoflex) according to the manufacturer’s protocol. Briefly, both floating and attached cells were pooled, washed, and resuspended in binding buffer. Fluorescein isothiocyanate-conjugated annexin V and PI were then added to the suspended cells at a ratio of 1:60 and the reaction was incubated in the dark for 15 min. Flow cytometric analysis was performed by analyzing gated cells.

### Quantitative real-time PCR

Total RNA was extracted with TRIzol reagent (Takara) according to the manufacturer’s instructions. cDNA was synthesized using PrimeScript RT Master Mix (Takara) according to the protocol recommended by the manufacturer. mRNA levels were determined by quantitative real-time PCR (RT-qPCR) using SYBR Premix Ex Taq (Takara). All reactions were performed in triplicate and the mRNA level of the housekeeping gene *GAPDH* was used as an endogenous reference control. The primer set targeting Cav-1 included the forward primer 5′-AACATCTACAAGCCCAACAACAAGG-3′ and the reverse primer 5′-GGTTCTGCAATCACATCTTCAAAGTC-3′. The primer set targeting Keap1 included the forward primer 5′-CAGAGGTGGTGGTGTTGCTTAT-3′ and the reverse primer 5′-AGCTCGTTCATGATGCCAAAG-3′. The primer set targeting GAPDH included the forward primer 5′-TGGGCTACACTGAGCACCAG-3′ and the reverse primer 5′-AAGTGGTCGTTGAGGGCAAT-3′. The data were analyzed relative to controls. All assays were performed on an ABI 7300 system (Applied Biosystems).

### Confocal microscopy

Cells seeded on coverslips were incubated with AFB1 (40 μm) at 37 °C for the indicated times. The cells were fixed in 4% paraformaldehyde for 20 min and permeabilized with 0.1% Triton X-100 at room temperature. The cells were then blocked in 5% bovine serum albumin and incubated with the Cav-1 antibody at a dilution of 1:1000 at room temperature for 2 h. After three washes with PBS, the cells were incubated with Alexa Fluor 488-conjugated secondary antibodies for 1 h. The cells were washed three times again with PBS and the nuclei were stained with 4′,6-diamidino-2-phenyl-indole (Roche) for 10 min. Images were obtained using a confocal laser scanning microscope (Zeiss LSM710 Meta, Carl Zeiss). The fluorescence intensity of the images was processed and quantified using ZEN Light Edition software (Carl Zeiss).

For autophagic marker detection, cells seeded on coverslips were washed with culture medium and then incubated at 37 °C for 30 min with 250 μl of 1 μmol/l DALGreen working solution. After a 6 h incubation with fresh culture medium, the cells were washed with Hanks’ HEPES buffer twice and DALGreen was observed by a confocal laser scanning microscope. The images were processed and analyzed using ZEN Light Edition software.

### siRNA transfection

siRNA transfections were performed according to the manufacturer’s instructions using the FECT transfection reagent (Dharmacon). Cells were seeded at a density of 4 × 10^4^ cells/cm^2^ in plate wells. The following day, cells were transfected with 100 nM Cav-1 siRNA/non-targeting siRNA mixed with Opti-MEM (Invitrogen) and incubated at 37 °C for 72 h, to ensure effective gene knockdown. Each siRNA transfection was performed in triplicate. Knockdown levels were monitored by western blotting at 72 h post transfection.

### Cell transfection and transient expression

For plasmid overexpression, cells were transfected as described previously^60^. Briefly, cells were seeded in 24-well tissue culture plates and grown overnight until they reached 75% confluency. Next, 0.8 μg of the Cav-1/vector plasmid construct was mixed with 50 μl of Opti-MEM (Invitrogen) for 5 min at room temperature. The mixture was then added to 50 μl of Opti-MEM containing 2 μl of Lipofectamine 3000 (Invitrogen) that had undergone similar incubation conditions. After a further incubation period of 20 min, the DNA–liposome complexes were added to the cells, which had been starved in Opti-MEM for 4 h before transfection. After 6 h of incubation at 37 °C, 1 ml of maintenance medium was added and the mixture was incubated for an additional 48 h. Each plasmid transfection was performed in triplicate. The expression levels were monitored by western blotting.

### Immunoprecipitation and western blotting

Immunoprecipitation and western blotting were performed essentially as described previously^61^. Cells were washed with cold PBS and then lysed in RIPA buffer (Pierce). The concentrations of proteins were measured by BCA protein assay kits. Equal amounts of protein were separated by 10% sodium dodecyl sulfate-polyacrylamide gel electrophoresis (SDS-PAGE). The proteins were then transferred to polyvinylidene fluoride membranes (Bio-Rad). The membranes were blocked with 5% nonfat milk in 1 × Tris-buffered saline with 1% Tween-20 (SCR) for 2 h at room temperature. Then, the membranes were incubated with the indicated primary antibodies at 4 °C for 16 h. After rinsing, each membrane was incubated with secondary antibodies conjugated with HRP. Immunoreactive bands were detected with ECL Plus enhanced chemiluminescence western blotting detection reagents (PerkinElmer Life Sciences). The protein bands were scanned and quantified based on optical densities using ImageJ (version 1.34 s) and normalized to GAPDH. Briefly, the obtained images were converted to 8-bit format. After conversion, the background was subtracted through the rolling ball radius method. Each band was individually selected and circumscribed with the rectangular ROI selection and “Gels” function, followed by quantification of peak area of obtained histograms. The values shown are the means of three independent experiments.

### Statistical analysis

Statistical analysis was performed using SPSS21.0 software. The experimental data are reported as the mean ± SD. Significance was determined with the two-tailed Student’s *t*-test for comparison of two groups. Significance between multiple groups was determined by analyses of variance and statistical significance (*P* < 0.05) is indicated by an (*) asterisk. In addition, *P* < 0.01 and *P* < 0.001 are marked with two (**) and three (***) asterisks, respectively, in the figures.

## Supplementary information

Supplementary Figure Legends

Supplementary Fig S1

Supplementary Fig S2

Supplementary Fig S3
