# Supplementary material for: Critical role of caveolin-1 in aflatoxin B1-induced hepatotoxicity via the regulation of oxidation and autophagy
Source: Cell Death Dis. 2020 Jan 2;11(1):6. doi: 10.1038/s41419-019-2197-6 (PMC6952418; doi:10.1038/s41419-019-2197-6)
Supplement: Supplementary file 1 — Supplementary Figure Legends [file 41419_2019_2197_MOESM1_ESM.docx]

**Supplementary Figure S1**

**Figure S1.** L02 cells transfected with NT, Cav-1 siRNA, Cav-1 plasmid and the vector control were untreated or treated with AFB1 (40 μM) for 36 h, Keap1 mRNA levels were determined by RT-qPCR. The data are shown as the mean ± SD of three independent experiments. *, P < 0.05; **, P < 0.01; and ***, P < 0.001.

**Supplementary Figure S2**

**Figure S2.** (A) L02 cells transfected with Cav-1 siRNA or NT siRNA were untreated or treated with AFB1 (40 μM). The protein levels of beclin-1, ATG5 and ATG7 were determined by western blot at 36 h after AFB1 treatment. (B) Cells transfected with GFP-tagged constructs expressing Cav-1 or the empty vector were mock-treated or treated with AFB1 (40 μM) for 36 h. The protein levels of beclin-1, ATG5 and ATG7 were determined by western blot. GAPDH served as a control for equal sample loading. The relative quantification of the detected signal was analyzed using ImageJ software and normalized to GAPDH. The data are shown as the mean ± SD of three independent experiments. *, P < 0.05; **, P < 0.01; and ***, P < 0.001.

**Supplementary Figure S3**

**Cav-1 shows a similar effect in different types of hepatocellular carcinoma cells**

To determine whether Cav-1 plays a similar role in other human hepatocytes, human hepatocellular carcinoma Huh7 cells and HepG2 cells were chosen for investigation. The effect of Cav-1 depletion on AFB1-induced hepatotoxicity was assessed in these cells. The cells were transfected with Cav-1 siRNA and then treated with AFB1. Downregulation of Cav-1 in these cells was confirmed by western blot (Fig. S3A). As shown in Fig. S3B & C, Cav-1 depletion significantly inhibited the AFB1-induced cell viability decrease and apoptosis in these cells. These results are consistent with those observed in L02 cells. Furthermore, similar results were obtained when ROS (Fig. S3D), MDA levels (Fig. S3E) and LC3 levels (Fig. S3F) were detected in Cav-1-depleted cells. These results indicate that Cav-1 has a similar effect in different types of hepatocellular carcinoma cells.

**Figure S3.** Huh7 and HepG2 cells were separately transfected with Cav-1 siRNA (100 nM) or NT siRNA (100 nM) for 72 h. (A) The reduction of Cav-1 protein levels after treatment with siRNA was detected by western blot, quantitated by densitometric analysis using ImageJ software and normalized to GAPDH. Following treatment with AFB1 (40 μM) for 36 h, the cell viability was then detected by CCK-8 assay (B), the induction of apoptosis was determined by annexin V/PI flow cytometry (C). ROS production was detected by DCF probe staining (D), MDA levels were measured by a detection kit (E), and the protein levels of LC3-I and LC3-II were determined by western blot at 36 h after AFB1 treatment (F). The data are shown as the mean ± SD of three independent experiments. *, P < 0.05; **, P < 0.01; and ***, P < 0.001.
