# Supplementary figures and images for: Critical role of caveolin-1 in aflatoxin B1-induced hepatotoxicity via the regulation of oxidation and autophagy
Source: Cell Death Dis. 2020 Jan 2;11(1):6. doi: 10.1038/s41419-019-2197-6 (PMC6952418; doi:10.1038/s41419-019-2197-6)

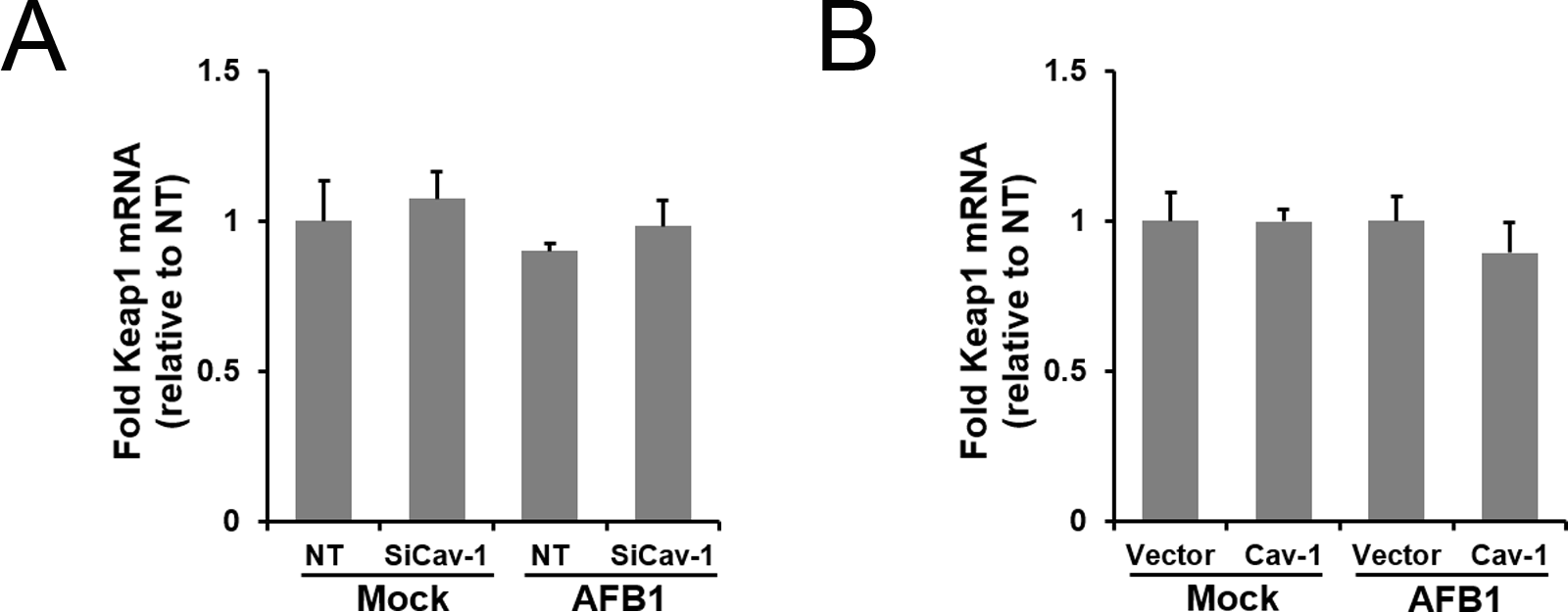

Supplement: Supplementary file 2 — Supplementary Fig S1 [file 41419_2019_2197_MOESM2_ESM.tif]

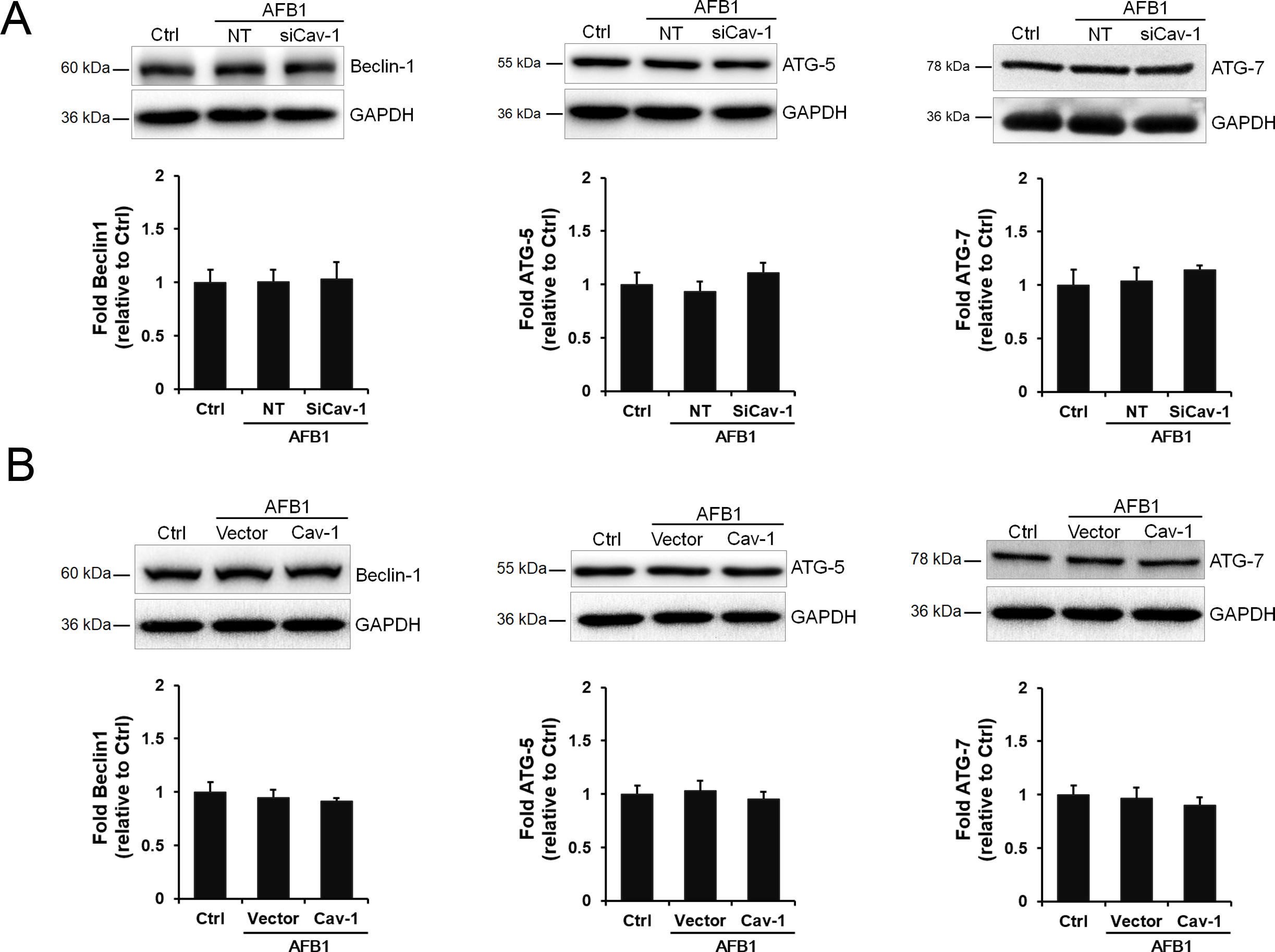

Supplement: Supplementary file 3 — Supplementary Fig S2 [file 41419_2019_2197_MOESM3_ESM.tif]

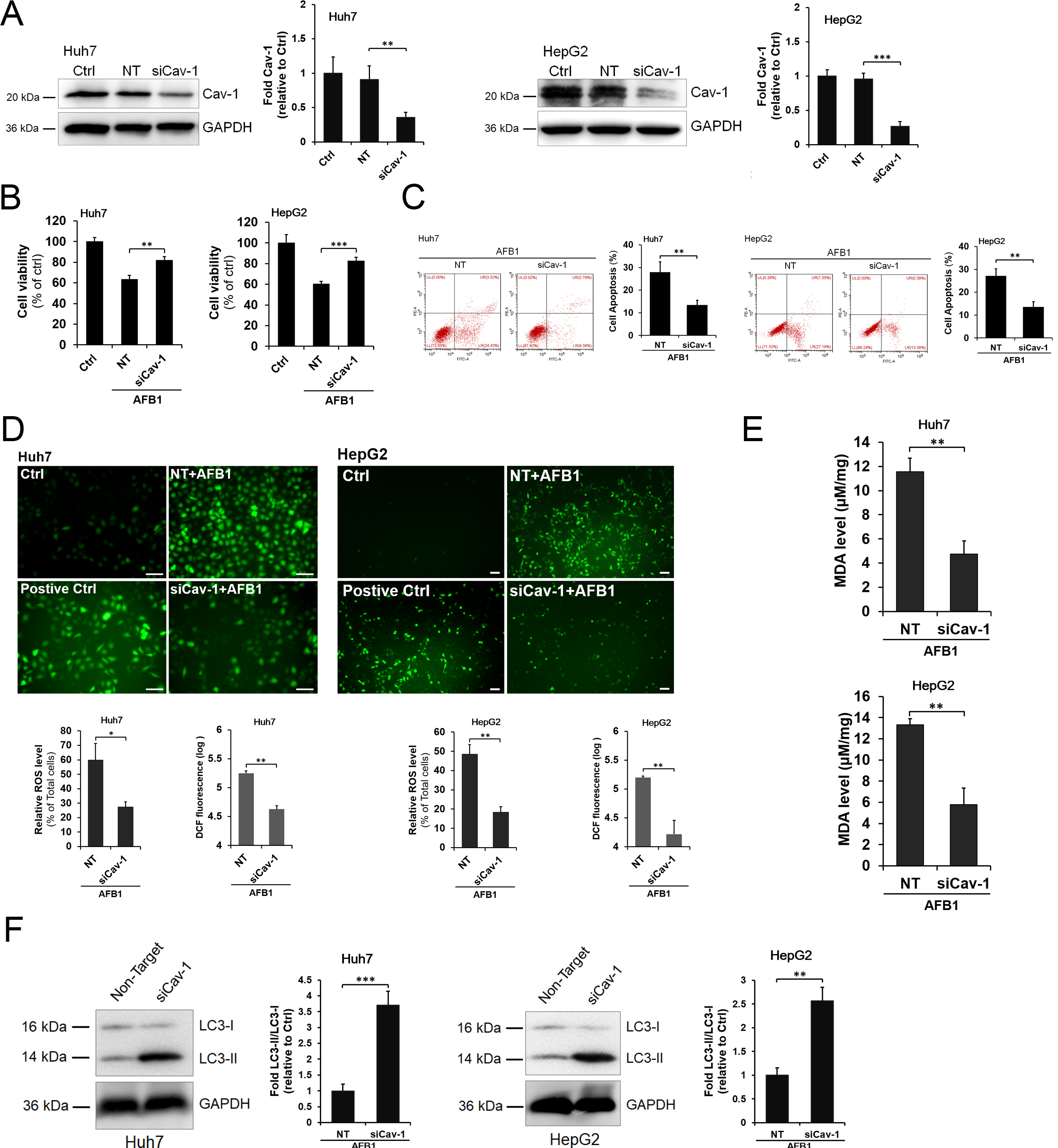

Supplement: Supplementary file 4 — Supplementary Fig S3 [file 41419_2019_2197_MOESM4_ESM.tif]
